# Supplementary material for: Synergistic Effects of Korean Mistletoe and Apple Peel Extracts on Muscle Strength and Endurance
Source: Nutrients. 2024 Sep 26;16(19):3255. doi: 10.3390/nu16193255 (PMC11478607; doi:10.3390/nu16193255)
Supplement: Supplementary file 1 [file nutrients-16-03255-s001.zip › nutrients-3219843-supplementary.pdf]

Table S1. Primer sequences for qPCR

Gene	Forward Primer 5′-3′	Reverse Primer 5′-3′
AMPK	AGAGGGCCGCAATAAAAGAT	TGTTGTACAGGCAGCTGAGG
PGC1*a*	GTCAACAGCAAAAGCCACAA	TCTGGGGTCAGAGGAAGAGA
SIRT1	TGTGAAGTTACTGCAGGAGTGTAAA	GCATAGATACCGTCTCTTGATCTGAA
TFAM	AAGGGAATGGGAAAGGTAGAG	ACAGGACATGGAAAGCAGATTA
MEF2C	CACCGGAACGAATTCCACTC	ATGCGCTTGACTGAAGGAC
DRP1	GGGCACTTAAATTGGGCTCC	TGTATTCTGTTGGCGTGGAAC
FIS1	GGCTGTCTCCAAGTCCAAATC	GGAGAAAAGGGAAGGCGATG
MFN1	TATCGATGCCTTGCGGAGAT	GGCGAATCACAACACTTCCA
MFN2	GGAGACCAACAAGGACTGGA	TGCACAGTGACTTTCAACCG
TNFa	CACAAGATGCTGGGACAGTGA	GAGGCTCCAGTGAATTCGGA
NF-κB	GCTGCCAAAGAAGGACACGACA	GGCAGGCTATTGCTCATCACAG
FOXO1	GCGGGCTGGAAGAATTCAAT	TCCTTCATTCTGCACTCGAATAAACT
MuRF1	ACCTGCTGGTGGAAAACATC	CTTCGTGTTCCTTGCACATC
Atrogin-1	AGGAGCGCCATGGATACTGT	GAAGTTCTTTTGGGCGATGC
MTSN	TGCAAAATTGGCTCAAACAG	GCAGTCAAGCCCAAAGTCTC
MyoD	GACAGGGAGGAGGGGTAGAG	TGCTGTCTCAAAGGAGCAGA
AKT	GCCCTCAAGTACTCATTCCAG	ACACAATCTCCGCACCATAG
4EBP1	CGGAAGATAAGCGGGCAG	CAGTGTCTGCCTGGTATGAG
S6K	TGAGTCAAGCCTTGGTCGAG	AAGAGTCGAGAGAGACGCCC
GAPDH	ACCACAGTCCATGCCATCAC	TCCACCACCCTGTTGCTGTA
